# Nutrient Utilization, Requirements and Nutrigenomics in Sheep and Goats

**DOI:** 10.3390/ani16050800

**Published:** 2026-03-04

**Authors:** Christopher D. Lu

**Affiliations:** College of Agriculture and Natural Resources Management, University of Hawaii, Hilo, HI 96720, USA; chrislu@hawaii.edu

**Keywords:** sheep, goat, nutrient requirement, nutrient utilization, nutrigenomics

## Abstract

The most recent National Research Council Nutrient Requirements for Sheep and Goats was published in 2007, one of the most consequential nutrient requirement recommendations for sheep and goats in the world. The enhancement of production efficiency, minimization of carbon footprint, and maximization of resource economy, among others, motivate the continuing discussion of nutrient utilization and refinement of nutrient requirements in sheep and goats that are increasingly important in various parts of the world. Progress has been made in the estimation of energy and protein requirements in sheep and goats, mainly utilizing empirical feeding experimentation, comparative slaughter techniques and minimum endogenous loss methods. In sheep, newer estimates of energy and protein requirements for maintenance and growth and partial efficiencies has been reported since 2007. There were suggestions that energy and protein requirements could have been affected by breed, wool growth, gender and size, with these reported values being similar or lower than the recommended values in international feeding systems such as NRC, ARC, INRA and AFRC. In goats, energy and protein requirements for growing goats were reported to be either higher or lower than the established recommendations, depending upon meat or dairy breeds. Effect of gender on energy requirement appeared to be related to the stage of growth or degree of maturity. Newer data also suggested that existing recommendations on nutrient requirements may not be adequate for non-pregnant and non-lactating pubertal females. In multiparous pregnant goats, energy and protein requirements for maintenance did not appear to be affected by days of pregnancy, but efficiencies of metabolizable energy and metabolizable protein utilization for pregnancy were. There were suggestions that metabolizable protein can be predicted from energy intake using equations that encompass both sheep and goats, but more data on goats were called for to account for specific differences in nutrition. In addition to sulfur, there has been progress made on the estimation of maintenance and growth requirements of calcium, phosphorus, potassium and magnesium in goats, with suggestions on the consideration of gender and breed differences. While conventional factors such as breeds and species, genotype, stage of maturity, gender, body composition, mobilization of tissue energy for production, and additional activity energy required due to resource limitation and acclimatization remain as important considerations for the estimation of nutrient requirements in sheep and goats, emerging factors such as climate change, heat stress, parasitism and secondary plant compounds that can affect nutrient utilization should also be considered in the estimation of nutrient requirements. Model equations and partial efficiencies used by NRC to predict energy and protein requirements for maintenance, growth, lactation, and fiber have been highlighted and discussed for the purpose of a more focus discussion and refinement for the future. Potential limitations of both traditional and emerging methodologies in determining the nutrient requirements in sheep and goats were discussed. The advancement in nutrigenomics can potentially move nutrient requirements beyond its population-based guidelines. To justify the research investment, emerging methodologies such as nutrigenomics will have to be linked more directly to the improvement of production efficiency via more precise prediction of nutrient requirement. With the assistance of artificial intelligence and more data obtained from sensor technology, precision nutrition has the potential to deliver nutrients precisely to individual animals and meet nutrient requirements in sheep and goats.

## Simple Summary

History, progress and pertinent issues of nutrient requirements of sheep and goats were presented and discussed. Mathematical models and conversion efficiencies used by the National Research Council to predict nutrient requirements in sheep and goats have been highlighted to persuade further research and discussion for the purpose of refinement of these nutritional standards. Nutrigenomics, artificial intelligence, precision nutrition, and other advanced methodologies were identified for their implications in future development and a more precise estimation of nutrient requirements in sheep and goats.

## 1. Introduction

The economic and social impacts of sheep and goat production in the improvement of living standards and alleviation of poverty in rural communities are recognized throughout the world [1]. Further understanding of nutrient utilization by sheep and goats contributes to the enhancement of production efficiency that can subsequently minimize carbon footprint and potentially conserve increasingly limited resources [2]. Requirements and utilization of structural and nonstructural carbohydrates, degradable and bypass proteins, lipids, minerals, and vitamins are essential parts of this important scientific understanding. Nutrient requirements are affected by multiple factors such as individuality, genetics, climates, diet, age, and physiological stages [3], and a continuous update of knowledge is essential. Relatively recent advancements in nutrigenomics [4,5,6,7] have led to improved knowledge on the molecular interaction between nutrients and other dietary bioactive components with respect to the genome, effect of food constituents on gene expression, and influence of genetic variation on nutrition, therefore contributing to a better understanding of nutrient utilization and requirements. Such understanding allows the optimization and customization of nutrition with respect to a subject’s genotype and offers great potential for a more precise determination of nutrient requirements in sheep and goats. The most recent National Research Council (NRC) Nutrient Requirements for Sheep and Goats was published in 2007 [3]. The scope of the Special Issue on “Nutrient utilization, requirements, and nutrigenomics in sheep and goats” is to contribute a better understanding of nutrient utilization and a more precise estimation of nutrient requirements in sheep and goats. This paper serves as an introductory article and provides an overview of the progress in establishing nutritional standards through traditional research in nutrient utilization and nutrient requirements, also considering new approaches such as precision nutrition and nutrigenomics, with the purpose of persuading further research and discussion and eventual improvement in the precision and application of nutrition standards to sheep and goat populations over a wide range of production systems and climatic conditions.

## 2. National Research Council and History of Nutrient Requirements in Sheep and Goats

The National Research Council was organized in 1916 under a congressional charter requested by then President Wilson of the United States [8]. It is the operating arm of the National Academy of Sciences, National Academy of Engineering, and Institute of Medicine, with the purposes of improving government decision and public policy, increasing public understanding, and disseminating knowledge in science, engineering, technology and health. The National Research Council supports studies in six divisions including Earth and Live Sciences. The Division of Earth and Live Sciences conducted studies and published “Nutrient Requirements of Animals”. Under the Division of Earth and Live Sciences, the Board on Agriculture and Natural Resources approves nutrient requirements of various animal species upon the recommendation of the Committee on Nutrition and Subcommittee of Nutrition of various animal species.

The 1st Edition of Nutrient Allowances for Sheep was published by NRC in 1945 [9] and was revised in 1949 [10], 1957 [11], 1964 [12], 1968 [13], and 1975 [14], with the 6th revised edition published in 1985 [15]. The 6th revised edition [15] was the last set of published NRC recommendations solely focusing on sheep. The 1st Edition of Nutrient Requirements of Goats was published by NRC in 1981 [16]. Since the NRC published its 1st Edition of Nutrient Requirements of Goats, there have been advancements in research pertaining to energy, protein [17,18,19,20,21,22] and sulfur [23,24,25,26,27,28,29] requirements in milk and meat- and fiber-producing goats that may or may not have been considered fully in the most recent NRC publication on nutrient requirements of goats [3]. A series of articles pertaining to energy and protein requirements in goats [30,31,32,33,34] were published prior to and considered in the 2007 NRC publication [3]. The latest version of nutrient requirements for sheep and goats combinedly appeared in “Nutrient Requirements of Small Ruminants: Sheep, Goats, Cervids and New World Camelids” and was published sixteen years ago [3]. Incorporated in the 2007 publication were the concepts of ruminally undegraded protein and microbial crude protein as sources of metabolizable protein (MP). For large ruminants, “Nutrient Requirements of Beef Cattle” and “Nutrient Requirements of Dairy Cattle” have been revised and published more recently [35,36]. Because of the intertwined assumptions and history of extrapolation, some of the refinements in nutrient requirement in these recent editions for large ruminants are relevant and can be considered as important references for the future edition of NRC requirements in small ruminants. The need to revise the almost two-decade old recommendation by NRC for sheep and goats should be considered. “Nutrient Requirements of Animals” is considered one of the most important nutritional standards published. They are commonly used as standards for future animal experiments, and NRC recommendations are among the most cited publications in animal nutrition. They can be considered as the culmination of nutritional accomplishment at a particular time period.

In addition to NRC, there are other notable international nutrition standards for sheep and goats that have been recommended by the Agricultural Food and Research Council in the United Kingdom [37,38,39,40], the Instituto National de la Recherche Agronomique in France [41,42,43], and the Commonwealth Scientific and Industrial Research Organization in Australia [44,45]. Most of these recommendations predated the most recent NRC recommendation for sheep and goats [3]. Nutrient requirements of meat-type sheep and goats were revised in China, after a decade-long coordinated national effort utilizing comparative slaughter trial, digestibility trial, and carbon–nitrogen balance technique [46].

## 3. Energy and Protein Requirements in Sheep

Since 2007, there has been progress made in the estimation of energy and protein requirements for maintenance and growth in sheep. Net energy requirement for maintenance (NE_m_) was 20% lower than NRC recommendations in a comparative slaughter experiment utilizing Texel crossbred lambs [47]. To estimate protein requirements by endogenous nitrogen (N) loss in Texel crossbred lambs [48], the growth pattern of the wool was found to influence protein requirements, and the estimated growth requirements were lower than those reported by the most nutritional systems. Salah et al. [49] conducted a meta-analysis study of 590 publications. With little or no difference in species, their [49] main conclusion was that ruminants including sheep and goats raised in tropical and warm areas had higher energy and protein requirements than those recommended by NRC, ARC, INRA and AFRC. Perhaps higher maintenance requirement in sheep and goats in those areas could be attributable to heat stress and extensive activities in the search of feed and water [49]. Comparative slaughter and digestion trials were conducted on Dorper × Hu crossbred lambs to determine energy and protein requirements [50]. Energy and protein requirement for growth in growing lambs were founded to similar to the NRC recommendations for early and later-maturating growing sheep. Oliveira et al. [51] suggested that total metabolizable energy (ME) and metabolizable protein (MP) requirements were lower than those recommended by the NRC and AFRC from a meta-analysis of seven experiments involving 243 sheep and proposed new equations for nutrient requirements of hair sheep raised in tropical regions. A hair sheep study [52] suggested that tables pertaining to energy and protein requirements recommended by international committees should be updated to include breeds raised in tropical regions. Their suggestions also raise the issue of recognizing the possible difference in energy and protein requirements between wool and hair sheep.

Pereira et al. [53] conduct a comparative slaughter experiment to evaluate the energy and protein requirements of intact male and castrated male and female Morada Nova lambs. There were indications that higher metabolizable energy efficiency utilization for gain in intact males contributed to lower net energy for growth (NE_g_). Lower net protein for growth (NP_g_) in females suggested a difference in sexes. Mendes et al. [54] utilized crossbreed Doper × Santa Ines lambs in a slaughter experiment to estimate energy and protein requirements for maintenance and growth and concluded that NE_m_ was similar to those recommended by the international committees, but NE_g_ was lower. The study confirmed the adequacy of current standards for energy requirement for maintenance, but perhaps the energy requirement for growth merits a revisit.

## 4. Energy and Protein Requirements in Goats

Since 2007, there have been a number of articles published pertaining to energy and protein requirements in goats. These studies indicated a need to recognize breed differences, body composition, including additional data directly from goats, and replace extrapolated data from other ruminant species. Net energy and protein requirements for growth and N requirement for maintenance in Boer × Saanen crossbred meat goats were suggested to be higher than previously published values for dairy goats [55]. When goats were included in a mechanistic model [56], termed the Cornell Net Carbohydrate and Protein System (CNCPS-S) which predicts nutrient requirements and biological values of feeds for sheep, Tedeschi et al. [57] suggested more data for goats are needed to account for specific differences in nutrition. In a slaughter experiment on Saanen male kids [58], energy requirements were reported to be lower than the requirements recommended by AFRC [40] and NRC [3].

While the energy requirement for maintenance appeared to be similar among male, female and male castrated goat kids in early growing stages [59], the energy requirement for growth could be affected by sexes at later stages of growth [60]. As body weight increased, the energy requirement per unit of metabolic body weight increased while that of protein requirement decreased [61]. During the postweaning growth period, NE_g_ were higher in castrated males and females than intact males, reflecting the difference in compositional gain [62]. The increase in NE_g_ was profoundly related to the stage of growth in non-pregnant and non-lactating pubertal female Saanen goats [63]. This could be attributed to the change in composition of gain as growth proceeded. These studies pointed out the inadequacy of the current recommendation by all major feeding systems for not taking into account the change in energy requirement during the entire growth phase. Almeida et al. [64] proposed the possibility of using body composition to predict maturity as the mature weight is known to affect protein and energy requirements in goats.

Teixeira et al. [65] concluded that maintenance requirements for F1 Boer × Saanen goat kids were greater than published values, and growth requirements were driven by efficiencies of deposition and largely dependent upon changes in body composition. In multiparous pregnant goats, the net energy for maintenance (NE_m_) and net protein for maintenance (NP_m_) for pregnancy did not change with days of pregnancy (DOP) [66]. However, both ME and MP utilization for pregnancy (k_p_) increased with the progress of pregnancy. It seemed that degree of maturity rather than sex affected energy requirements for maintenance in growing dairy goats, but the energy utilization for growth was lower in males than females and castrated males [67]. This is another indication of the effect of compositional gain on energy requirement as one would expect more fat deposition as the stage of growth progresses. A new equation for the prediction of NP_m_ was proposed based on the conclusion from a meta-analysis that sex did not affect either the protein requirement for maintenance or efficiency of protein utilization [68].

Santos et al. [69] proposed prediction equations to estimate microbial protein synthesis to be used for the calculation of rumen degradable protein (RDP) requirements from MP in sheep and goats. Because sheep and goats have similar metabolizable crude protein (MCP) efficiency, combined equations from sheep and goats can be used to predict MCP synthesis in the rumen from energy intake. With the Akaike Information Criterion Index ranged from 2755 to 3007, they proposed that the prediction could be based on total digestible nutrients (TDN), digestible organic matter (DOM), or ME intake by the following equations: MCP (g/day) = 12.7311 + 59.2956 × TDN intake; MCP (g/day) = 15.7764 + 62.2612 × DOM intake; and MCP (g/day) = 12.7311 + 15.3000 × ME intake. These prediction equations could contribute to the refinement of the protein requirement based on metabolizable protein rather than crude protein in sheep and goats.

## 5. Macromineral Requirements in Sheep and Goats

One major improvement in recommending macromineral requirements for sheep and goats by NRC [3] is that the distinction between species with more data directly from goats was included, instead of solely relying on extrapolation. INRA [70] provided more detailed information and used more complex equations, and the recommended values were sometimes different. Both systems consider physiological functions such as growth, pregnancy, lactation, breed, sex, age, and physiological stage within the function. There were newer studies reported on macromineral requirements in sheep and goats since 2007. Calcium requirements for maintenance and growth in wool sheep were recently recommended [71]. Effect of sex on net maintenance requirements of calcium (NCa_m_), phosphorus (NPh_m_), potassium (NK_m_), and magnesium (NMg_m_) in dairy goats (5–45 kg BW) were evaluated from a meta-analysis based on an almost equal number of animals in comparative slaughter experiments and in minimum endogenous loss experiments [72]. Based on the data from comparative slaughter experiments, sex did not affect NCa_m_, NPh_m_, or NK_m_, but NMg_m_ of intact males was greater than castrated males or females. Similar observations were derived from minimum endogenous losses experiments with the exception of higher NMg_m_ in intact males. The meta-analysis pointed out that NPh_m_ was significantly lower than the current recommended values [3] and merits further evaluation. Lower P requirement may reflect to the more efficient P recycling in goats. The newer version of INRA [70] has made adjustments to recommend a lower P requirement. Net requirement of Ca, P, and Mg for growth (NCa_g_, NPh_g_, or NK_g_, and NMg_g_) in meat goats (Boer crossbred) increased with stage of growth, while those for Na and K (NNa_g_ and NK_g_) decreased, suggesting the idea to consider genotype, and more data may be needed for goats [73]. The Ca, P, Mg, K, and Na requirements of Saanen goats were estimated from two experiments (n = 75 in comparative slaughter experiment, and n = 58 in growth trial) [74]. They reported that the daily net macromineral requirements for maintenance did not differ among the sexes. For growth, NCa_g_, NPh_g_, and NK_g_ were not different among the sexes, but sex could affect NNa_g_ and NK_g_ [74]. It is not clear why sex affects net requirement of K and Na but not Ca or P.

It appears that there is an interaction between sex and stage of maturity in net requirements of several macrominerals in dairy goats. Vargas et al. [75] compiled data from six comparative slaughter experiments and analyzed the effects of sex and stage of maturity on NCa, NP_g_, NNa_g_, NK_g_, and NMg_g_ in Saanen goats from 5 to 45 kg BW. For improving the accuracy of micromineral requirements in dairy goats, the maturity stage across sexes should be considered [73]. There were indications that mineral requirements may be different between sheep and goats. In the overview of Ca and P utilization between sheep and goats, Wilkens et al. [76] concluded that dietary supply of Ca did not affect ruminal Ca absorption and renal excretion in both species. However, they observed that goats could compensate for low Ca availability while sheep could not, as evidenced by a smaller increase in plasma calcitriol with a greater increase in the concentration of a bone resorption marker in sheep than in goats. A greater salivary P secretion suggested more efficient recycling in goats. Responses to lactation through the increase in Ca absorption in the gastrointestinal tract and bone mobilization were similar in sheep and goats. Recognizing similarities and difference in Ca and P absorption and metabolism between sheep and goats can improve the precision of estimating requirements.

## 6. Micromineral Requirements in Sheep and Goats

Considering physiological stages like growth, pregnancy and lactation, NRC [3] recommended requirements for cobalt (Co), copper (Cu), iodine (I), iron (Fe), manganese (Mn), molybdenum (Mo), selenium (Se), and zinc (Zn) in sheep and goats. Age, sex and fiber production such as wool and mohair were also considered in certain microminerals. More specific requirements based on body weight or predict equations were listed for Cu, I, Fe, Mn, Se, and Zn. Potentially essential microminerals such as boron, chromium, and others were also mentioned. Although there are similarities in some of the micromineral requirements in sheep and goats, distinct differences in Cu were documented between these two species [77,78]. Sheep are susceptible to Cu toxicity while goats are more tolerant, resulting in a higher Cu requirement in goats. INRA [70], because of the inclusion of updated data, provides more specific recommendations, especially in goats. It recognized the higher needs of microminerals in early lactation and growth and recommended higher requirements in some microminerals such as Co, Mn and Zn, while others remain similar to NRC [3]. Bioavailability, delivery and mineral interactions seem to be the foci of more recent micromineral research, development and advancement [79,80,81,82]. The effect of micromineral interactions on bioavailability of certain micromineral merits further attention. Copper, sulfur and Mo interactions in goats have been reviewed [25] and could be referenced for future revision. The differences in bioavailability between organic forms, such as chelated and hydroxy microminerals, and traditionally used inorganic forms should be recognized. Micromineral requirements can be affected by heat stress [83] because of the increased need of certain minerals to mitigate oxidative stress and the disruption of mineral balance due to lower feed intake and higher excretion resulting from panting and sweating. The requirements for Cu, Se and Zn that play important roles in heat stress-related oxidative process as antioxidants are increased. Increases in micromineral requirements as a result of heat stress have implications in sheep and goat production in regions that are affected by climate change. Micromineral requirement in sheep and goats that consumed novel feed resources in developing regions has been recently discussed [84]. Because of the toxicity of certain microminerals, both minimum (requirement) and maximum (toxicity) are important considerations in ration formulation, and a ranged rather averaged value may be desirable.

## 7. Water Requirements

Water, needed to maintain basic physiological functions that underline productive performance, can be considered one of the most important nutrients for sheep and goat production. Water requirements can be met through free access to clean water. However, it can hinder optimal production when the access is limited due to drought, an arid environment, salinity, or when quality is compromised due to contamination [85,86,87]. Water requirements (mL/kg BW) differed in sheep for maintenance, pregnancy and lactation, with lactation being 2.8 times of that of maintenance [3]. NRC water requirements are less specific for goats and are not based on metabolic body weight (MBW) but related to dry matter intake (DMI). The water requirement is suggested to be 4 to 5 times DMI for lactation and 2 to 3 times DMI for maintenance. In INRA [70], general recommendations for water requirement related to DMI are provided for goats (3.3 L of water per Kg of DM intake) while they are less specific for sheep. In a more recent study, water intake of 5.6 L/day sustained 3.3 Kg/day of milk production in lactating alpine goats grazing under 13 to 27° C with a temperature–humidity index of 56 to 76 regardless of daytime water restriction [88]. Defining water requirement based on MBW is desirable, but a range rather than an average can be more practical because water intake is commonly known to be affected by environmental temperature, feed, physiological stages, and level of production, especially lactation. Increases in dietary protein or feed energy source (fat vs. starch) can affect water requirement to a lesser extent due to deamination and metabolic water production as parts of metabolic processes. Meeting water requirements can be more of a management issue rather than an issue directly related to ration formulation. A general recommendation considering MBW, DMI, stage of lactation, production level and environmental temperature may be helpful in meeting water requirements in sheep and goats under a range of climate and production systems.

## 8. Fiber Requirements

From the stimulation of papillary development in the rumen and prevention of acidosis to milk fat synthesis, fiber plays an essential role throughout the life span of sheep and goats. Traditionally, the fiber requirement is met by providing an optimal amount of crude fiber (CF), neutral detergent fiber (NDF) or acid detergent fiber (ADF) in the diet to maintain normal rumen function that is conducive for microbial degradation and synthesis. Eating, ruminating and total chewing time have been used as indicators for rumen function in sheep and goats of various physiological stages [89,90,91,92,93]. As a result of chewing and rumination, salivary flow, rate of digestion and rate of passage contribute to buffering capacity of rumen and facilitate nutrient digestion. Because of fill factors, free access to forage/roughage without concentrated supplements may limit production as frequently observed in sheep and goats consuming novel and lower quality of feed sources [84]. At least 10% crude fiber and 10 to 20% roughage is recommended for growth and maintenance in sheep [3]. Recommendations for fiber requirements are less specific for goats. INRA [70] suggests a minimum of 33–38% NDF for lactating ewes and 22 to 26% NDF for growing and finishing ewes. Their feed unit system took into account the physical fill of fiber. For lactating does, it suggests 18–20% ADF or 41% NDF and 23% ADF for growing goats. A recommendation of minimum fiber for sheep and goats of various physiological stages seems to be a reasonable approach and can be incorporated into ration formulation. As more quantitative information on chewing and rumination is available, it may be desirable to establish minimum fiber requirements based on MBW in sheep and goats of various physiological stages [94]. Furthermore, rumination time based on MBW in order to accommodate various factors such as particle length [95], forage-to-concentrate ratio [89], and physical fill associated with fiber digestion can also be considered.

## 9. Estimating Nutrient Requirements

### 9.1. Physiological Functions and Classifications

Maintenance, growth, lactation, pregnancy, activity, breeding, and fiber growth are major physiological and/or productive functions considered in the determination of nutrient requirements for sheep and goats. In sheep, NRC [3] classifications of nutrient requirements include mature ewes, yearling farm ewes, yearling range ewes, rams (maintenance and prebreeding), growing lambs and yearlings (early and late maturing). Mature ewes include four additional subclassifications: maintenance, breeding, gestation (early, late; single, twin and triplet), and lactation (early, mid, and late; single, twin, triplet or more) [3]. In goats, NRC classifications of nutrient requirements differentiate Angora goats from dairy and nondairy goats [3]. For dairy and non-dairy mature does, it is further classified as maintenance, growth, breeding, gestation (early, late; single, twin and triplet) and lactation (early, mid, and late; single, twin, triplet or more). For dairy and non-dairy mature bucks, it is classified as maintenance and prebreeding. For Angora goats, the classifications include growing Angora kids (male and female), mature Angora males (maintenance and breeding), and mature angora females (maintenance, breeding, gestation and lactation). Gestation is further classified as early and late gestation, fetal size (single, twin and triplet), and lactation including early (single, twin) and late lactation.

Intake level, genetic potential for productivity, composition of gain, mobilization of tissue energy, level of activity such as grazing, and heat and cold stress could affect nutrient requirements in sheep and goats [3]. Furthermore, parasitism can affect nutrient requirements of sheep and goats. It is known that nutrient requirements in sheep and goats can be influenced by the environment [96]. As the effect of climate changes on the environment intensifies, it is likely that precise estimation of nutrient requirements in sheep and goats will become even more important to minimized greenhouse gas emissions and carbon footprint by animals in the future.

### 9.2. Estimating Energy Requirements

**Figure 1:**
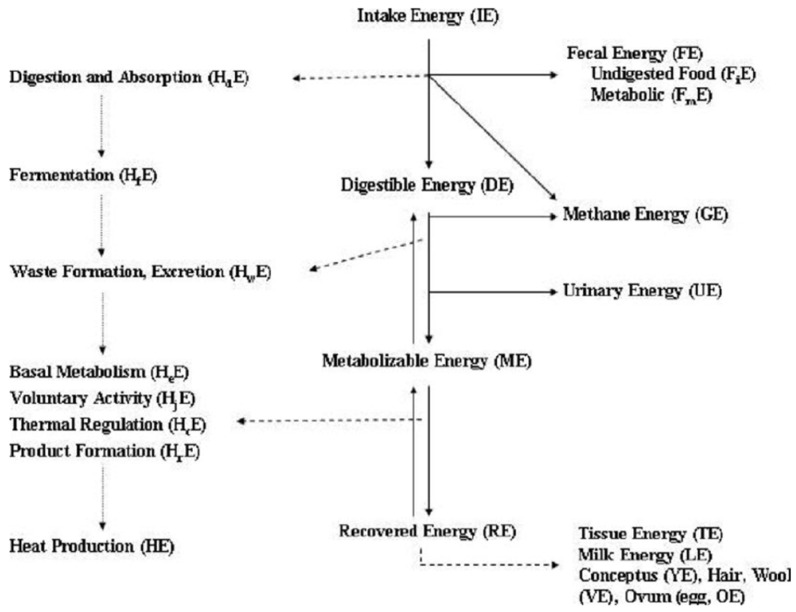
Schematic diagram of energy utilization by sheep and goats (modified from [98]).

Estimation of energy requirements in sheep and goats are based on two fundamental laws of thermodynamics and a portion of Einstein’s theory of relativity. Firstly, energy input must be equal to energy output plus or minus any changes in body energy (energy cannot be created or destroyed). Secondly, no transformation of energy is 100% efficient, and the inefficiency is lost as heat (entropy of the universe always increases). By applying these two principles, it affords the equivalence between mass and energy and allows the conversion of body mass (kg) to energy (calories). Energy utilization by ruminants including sheep and goats can be partitioned into intake energy (IE), digestible energy (DE), metabolizable energy (ME), and net energy (NE) (Figure 1) and has been described and discussed [97]. A number of conversions have been used in establishing energy requirements in sheep and goats [3]. Those include the following: DE (Mcal) = 4.4 TDN (kg), ME (Mcal) = DE (Mcal) × 0.82, estimate of heat production, conversion of ME to NE_M_, conversion of ME to NE_g_, conversion of ME to NE_L_, and 1 Mcal = 4.184 MJ.

### 9.3. NRC Equations for Predicting Energy Requirements for Sheep and Goats

A number of important equations have been used to estimate energy requirements in sheep [3]:

For maintenance, unadjusted:

0.062 Mcal NE/kg SBW^0.75^; (ME_m_ × 0.644 = NE_m_) (1)

For growth:

NE_g_, Mcal/d = ADG, kg × TEC_avg_; TEC_avg_, Mcal/kg = (TE_fin_ − TE_init_)/(FBW_fin_ − FBW_init_); (ME_g_ × 0.6 = NE_g_) (2)

For lactation:

NE_l_, Mcal/d = (251.73 + (89.64 × MFC, %) + (37.85 × (MPC, %)/0.95))) × 0.001 × MY, kg/d; (ME_l_ × 0.644 = NE_l_) (3)

A number of important equations have been used to estimate energy requirements in goats [3]:

For maintenance, unadjusted:

101–149 kcal ME/kg FBW^0.75^; (ME × 0.644 = NE) (4)

For growth:

ME_g_ = 3.20–6.81 kcal/g ADG; ME_tg_ = 8.89 kcal/g ADG (Angora); ME_f_ = 37.5 kcal/g ADG (Angora) (5)

For lactation:

ME_l_ = 1.25 Mcal/Kg 4%FCM (6)

where SBW is shrunk body weight, ME_m_ is metabolizable energy required for maintenance, NE_m_ is net energy for maintenance, NE_g_ is net energy for gain, ADG is average daily gain, TEC_avg_ is average energy concentration on a full body weight basis during the feeding period, TE_fin_ is final tissue energy, TE_init_ is initial tissue energy, FBW_fin_ final full body weight, FBW_init_ is initial full or unshrunk body weight, ME_g_ is metabolizble energy used for tissue gain, NE_l_ is net energy for lactation, MFC is milk fat concentration, MPC is milk true protein concentration, ME_l_ is metabolizable energy used for lactation, ME_tg_ is metabolizable energy used for nonfiber tissue gain, and FCM is fat-corrected milk.

### 9.4. Estimating Protein Requirements

**Figure 2:**
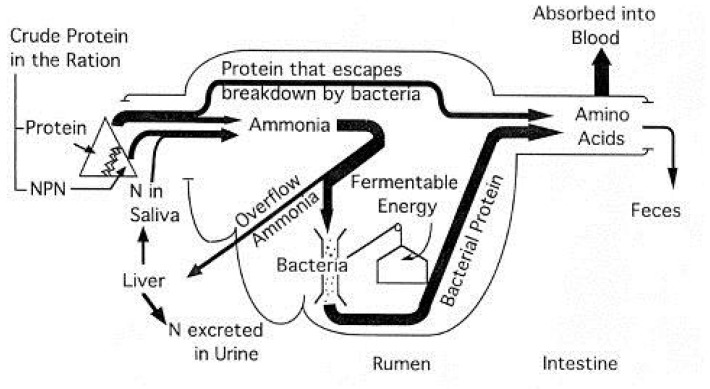
Protein digestion in sheep and goats (adapted from [78]).

Nitrogen utilization in ruminants has been described [98]. Protein digestion in sheep and goats (Figure 2) is largely adopted from the model developed by Satter and Roffler [99], although there may be small differences in digestion kinectics such as rumen turnover rate between small and large ruminants [100,101].

There are a number of assumptions used pertaining to the efficiency of conversion in the estimation of protein requirements in sheep and goats [3]. Efficiency of dietary MP for maintenance (K_pm_) is equal to 1.0. Efficiency of MP for maintenance (K_pm_) is similar for Metabolic Fecal Crude Protein (MFCP), Endogenous Urinary Crude Protein Loss (EUCP), and scurf CP. Range of K_pm_ from 0.67 to 1.00 is due to correction of MFCP for microbial CP. K_pm_ may vary with age and may be lower with greater intake. Efficiency of MP for growth (K_pg_) ranges from 0.5 [15] to 0.7 [102]. K_pg_ may vary with age, BW and body condition score. Efficiency of dietary MP for tissue gain in lactating goats is equal to 0.59. Efficiency of MP for lactation (K_pl_) varies: 0.58 [43], 0.59, 0.64 [33], 0.67 [103], 0.68 [39], and 0.7 [103]. Efficiency of mobilized tissue protein for lactation (K_pl-t_) is yet to be defined. Efficiency of mobilized MP for milk protein synthesis is equal to 0.69. Efficiency of MP for fiber growth (K_pf_) varies: 0.26 [39], 0.48 [104], 0.5 [15], and 0.6 [81]. K_pf_ may not be the same for wool sheep and angora goats and may be different between diet and mobilized tissue. Efficiency of mobilized MP for clean mohair fiber synthesis is equal to 0.61. Estimation of scurf and fiber crude protein requirements varied from 0.1125 g/kg BW^0.75^ [39] to 0.2 g/kg BW^0.60^ [103,105], as fiber production can be affected by season, breed, species, and frequency of shedding. Efficiency of MP for pregnancy and gestation (K_ppreg_) varies: 0.33 [34], 0.4 [43], 0.65 [106], 0.70 [102], and 0.85 [39,40]. K_ppreg_ may not be the same for sheep and goats and may be influenced by the development of mammary gland.

It is worth mentioning that evidence affecting the estimation of protein requirements has emerged. Plant secondary metabolites can decrease activities of intestinal enzymes and interact with the epithelium lining which may ultimately affect the estimation of protein requirements [3]. Internal parasites can result in greater use of AA for the synthesis of immunoglobulins and cytokines; increase replacement and repair of GI tract tissues with higher rates of liver protein synthesis can also affect the estimation of protein requirement [3].

### 9.5. NRC Equations for Predicting Protein Requirements in Sheep and Goats

A number of important equations have been used to estimate metabolizable protein requirements in sheep [3]:

For maintenance, plus fiber:

MP_m_, g/d = (SF − CP_E_/0.6) + (U − CP_E_/0.67) + (F − CP_E_/0.67) (7)

For growth:

MP_g_, g/d = NP_g_/0.7; NP_g_, g/d = ADG, g × TPF_r_ × 0.92 (8)

For lactation:

MP_l-d_, g/d = NP_l-d_/0.58: NP_l-d_, g/d = (10 × MTPC, % × MY, kg/d) − (ADG, g × TPF_r_) (9)

A number of important equations have been used to estimate protein requirements in goats [3]:

For maintenance, plus fiber:

MP_m_, g/d = MFCP + EUCP + (0.2 g/kg BW^0.60^) (10)

For growth:

MP_g_, g/d = 0.290 g/g ADG (Dairy and Indigenous); MP_g_, g/d = 0.404 g/g ADG (Meat); MP_g_, g/d = 0.281 g/g tissue gain (Angora) (11)

For lactation:

MP_l_ = 1.45 g/g (12)

For Fiber:

MP_clean mohair_ = 1.65 g/g (13)

where MP_m_ is metabolizable protein required for maintenance, SF − CP_E_ is scurf and fiber protein, U − CP_E_ is endogenous urinary crude protein, F − CP_E_ is metabolic fecal crude protein, MP_g_ is metabolizable protein available for gain and growth, NP_g_ is net protein for gain, ADG is average daily gain, TPF_r_ is protein concentration in tissue accreted or mobilized on an empty BW basis, MP_l-d_ is dietary metabolizable protein required for lactation, NP_l-d_ is net protein for lactation from the diet, MTPC is milk true protein concentration, MY is milk yield, and MP_l_ is metabolizable protein used for lactation.

Simplified energy and protein requirements have been tabulated (Table 1) for the purpose of comparison between sheep and goats. There are notable differences in both energy and protein requirements between these two species.

### 9.6. Amino Acid Requirements

Because of microbial degradation of dietary protein and nonprotein nitrogen, it is challenging to precisely quantify dietary amino acids requirements in sheep and goats. Rumen microbes degrade dietary protein and can alter dietary amino acid profiles. Instead, a system of metabolizable protein, the summation of microbial protein and undegraded dietary protein, has been used [3,70]. In general, the amino acid profile of microbial protein is favorable as it is similar to body and milk protein [107,108]. However, the quantity of microbial protein synthesis may not be sufficient for milk production and muscle growth especially in high-producing sheep and goats. Recent effort on amino acid requirements in sheep and goats is centered around the identification of limiting amino acids and delivering them directly to the small intestine for absorption [108,109]. Methionine and lysine are generally considered as limiting for milk production, muscle growth and wool growth. In addition, histidine and leucine can be limiting for wool growth. Supplementation of rumen-protected lysine and methionine improved weight gain and feed efficiency in sheep fed low-protein diets [109]. Fetal growth, neonatal survival rates and fetus brown adipose tissue were enhanced by intravenous administration of L-arginine to ewes [110]. Methionine, lysine and leucine are limiting in goats [111], and their rate of digestion could be faster in goats, suggesting that different protein/AA ratios may be required for sheep. Sulfur-containing amino acids like methionine and cysteine are high in keratins; therefore, they are particularly important for wool growth [112]. Arginine, histidine, leucine, and lysine requirements had been quantitatively suggested in South African Mutton Merino lambs [113,114]. Emerging issues on genetics [115], the environment [116], species [108], and balancing rather than increasing amino acid supply merit further discussion and research.

### 9.7. NRC vs. INRA Equations for Predicting Energy and Protein Requirements in Sheep and Goats

In the more recent versions of the French feeding system for sheep and goats [70,107], Unité Fourragère Lait (UFL, Milk Feed Unit), UFV (Unité Fourragère Viande, Meat Feed Unit) and Protéine Digetible dans l’Intestin (PDI, Protein Truly Digestible in the Small Intestine) were used. The UFL, derived from NE_L_ and a relative unit, is the net energy value for lactation equivalent to one Kg of air-dried barley (7.12 MJ of NE). It considered the advantages of on-farm familiarity and easy reference for producers. The PDI, a similar concept to NRC’s MP, is calculated from Protéines Digestibles dans l’Intestin grêle permises par l’Azote (PDIN, protein digested in the small intestine when nitrogen is the limiting factor) and Protéines Digestibles dans l’Intestin grêle permises par l’Énergie (PDIE, protein digested in the small intestine when energy is the limiting factor), with the lower amount used for ration formulation. The NRC [3] covers sheep, goats, deer, elk, caribou, alpacas, and llamas while INRA [117] covers cattle, sheep and goats. Recognizing the differences in species, a substantial number of studies directly from goats were utilized in NRC [3] to replace extrapolated from sheep and cattle from its previous version in 1981. While attempting to compare the precision of estimating nutrient requirements among various systems, one must recognize the geographic emphasis of each system. As an update to INRA [117], INRA [70] considered a wider range of climate variation and modified maintenance and growth requirements in sheep and goats, especially those raised in the tropics. For example, maintenance requirements in the tropics may be higher than those in a temperate climate. It also recognized the difference between species and sex. The calculation of fermentable organic matter was updated to better predict the production of volatile fatty acids in the rumen. Through the incorporation of wider environmental and species consideration, INRA [70] hopes to improve the precision of meeting nutrient requirements in sheep and goats under different production conditions and goals. While a more complex model may improve the precision of estimating nutrient requirements in a specific genotype and environmental condition, it is equally important to maintain user friendliness and practical application of a wider environmental condition and species. Because of the historical use of data from the regions, both INRA [70] and NRC [3] may underestimate the nutrient requirements of sheep and goats fed novel feed with lower nutritional qualities, especially those raised in warmer and tropical regions [84]. An update of nutrient requirements of goats in hot environments has been reviewed [118], suggesting the need to tailor/revise nutrient requirements for goats in hot climates.

## 10. Limitations in Establishing Nutritional Standards

Because of a number of factors that have been identified to influence the determination of nutrient requirements, it is apparent that nutritional standards are relative, not absolute. It is targeted towards a population but not to specific individuals or a subset of group of animals, unless it is well-defined at the beginning of the experiment. Nutritional standards can be determined through deduction or empirical methods. They are not always verified by additional animal experiments once established. These standards reflect the professional judgment or opinion of a group of experts during a particular period of time. In order to apply these standards effectively, the users must recognize the main sources of variation such as individual, genetic, climatic, dietary, age and physiological stages.

The basic methodology in the evaluation of nutrient requirements includes empirical and factorial approaches. The empirical method is based on observations or experience and is verifiable. The factorial method is related to a factor or integral parts of a factor. The minimal amount of nutrients needs to maximize population response for one or more performance criteria such as growth, and lactation in a defined period is usually determined by the empirical method [119]. Combining the estimated requirements for maintenance, growth and/or lactation, daily requirements of animals at a specific point or a period of time are normally achieved by the factorial approach. The empirical method estimates optimal nutrient allowances from a population perspective, whereas the factorial method estimates the needs of a reference animal during a very short period of time [119]. There is always a need to reconcile the difference in nutrient requirements between empirical and factorial methods.

## 11. Nutrigenomics and Nutrient Requirements

The implication of nutrigenomics on nutrient requirements lies in the linkage between an individual’s genetic makeup and its nutrient needs. Nutrigenomics is derived from molecular nutrition and focuses on the effect of nutrients on physiology and health at molecular and cellular levels [5]. Tools used in descriptive and mechanistic studies, such as system biology data integration, advanced biostatistics, imaging, calorimetry, cell biology, genomics with single nucleotide polymorphisms (SNPs) and epigenomics, transcriptomics, proteomics, and metabolomics, continue to provide insights for our understanding of important gene–diet interactions and lead to better meeting individual animals’ nutritional needs [5].

The determination of molecular mechanisms underlying animal nutrition, health and disease offers great potential for promoting health and lowering mortality and morbidity, contributing to the rise of the science of nutrigenomics [1]. Nutrigenomics is the study of the effect of food constituents on gene expression and is broadly defined as the relationship between nutrients, diet, and gene expression [4]. It can identify molecular interaction between nutrients and other dietary bioactives with the genome. It focuses on the influence of genetic variation on nutrition and correlate gene expression. With the potential to optimize and customize nutrition with respect to subject’s genotype, nutrigenomics is promising and can be useful for disease prevention, health promotion and anti-aging. Nutrigenomics can be considered as a part of broader molecular nutrition.

Nutrigenomics and other omics (genomics, epigenomics, transcriptomics, proteomics and metabolomics) and bioinformatic tools are poised to accelerate our understanding of regulation in small ruminants induced at multiple levels by dietary nutrients during their utilization for producing milk, meat, wool, or reproduction [6]. Initial data indicate that the nutrigenomics approach may eventually lead to more precise management of goats and sheep and improve the utilization of feed resources in a more optimal fashion [6], therefore affecting the estimation of nutrient requirements. Nature’s largest gene depository resides in bacteria. Rumen microbes are an integrated part of nutrient digestion and utilization in sheep and goats. Total genetic composition of rumen microbes consists of core genomes and accessory genomes. Separation of commonly shared genes (core genome) from strain-specific genes (accessory genome) will certainly contribute to a better understanding of microbial interaction with nutrients. Understanding microorganisms in the rumen through nutrigenomics can enhance the understanding of nutrient utilization and subsequently improve the precision of the determination of nutrient requirements in sheep and goats.

Nutrigenomics in sheep and goats is considered to be in its infancy, but it can potentially impact nutrient requirements of individual animals based on their genetic makeup. There are increasing reports related to nutrigenomics in sheep and goats [6,120,121,122,123,124], with most of them exploring how dietary composition influences gene expression with some implication of efficiency of nutrient utilization. The extent of the impact of continuous advancements in nutrigenomics on the precision of nutrient requirements has not been investigated, examined, or discussed. Because of the adaptation to the environment or other lesser known factors, mutated genes that are responsible for a more efficient utilization of nutrients can be identified. The identification of genes that have a direct effect of nutrient utilization and efficiency through SNP has the potential to alter the nutritional standards established from traditional empirical approaches. These genes with favorable variation can be selected within a breed or population, directly introduced through crossbreeding, or directly edited in a genome [125]. Through genetic selection or direct insertion of these genes, nutrient requirements which normally are targeted to the population could be affected because of the changes in genetics in the population. It is not yet clear how the eventual progression of nutrigenomics in sheep and goats will affect the methodologies in the estimation of nutrient requirements in changing animal populations.

Nutrigenomics can also lead to precision nutrition that is tailored to more specific needs for individual animals, therefore meeting individuals’ nutrient requirements. Nutrigenomics takes individual variations into account in order to better meet nutrient requirements for optimal production. Traditionally, the recommendations for nutrient requirements come in the form of population-based guidelines. The advancements in nutrigenomics can potentially move beyond the population-based guidelines. It is rooted in the notion that population guidelines that have been designed for all animals may not optimal for individuals. While nutrigenomics continues to advance, translating complex research into evidence-based practical guidelines remains a challenge. Its eventual impact on recommendations of nutrient requirements remains to be seen.

## 12. Artificial Intelligence, Precision Nutrition and Nutrient Requirements

In sheep and goat production, especially dealing with larger herd size and aiming to reduce our carbon footprint and increase the efficiency of production, the digitization and application of artificial intelligence (AI) have immense implications. From nutrition [126], livestock identification and management [127], health detection [128], and feeding behavior [129] to animal welfare [130], application of AI has the potential to revolutionize the management of large-scale small ruminant production. From machine learning to deep learning, AI models such as Artificial Neural Network, Convolutional Neural Networks, Adaptive Neural Fuzzy Inference System, Pattern Recognition, along with numerous sensors and devices have been used in animal production [131]. Precision nutrition is an area that can deliver nutrients precisely to individual animals and has potential to meet nutrient requirements for maintenance and production of meat, milk and fiber in sheep and goats. The application of AI has become an integral part of precision nutrition. The use AI tools such as Internet of things (IoT, mass data collection and storage), electronic identification (EID, ear tags that can be read by a handheld reader), and software of feed formulation, livestock management, reproductive optimization, and quality assessment makes it possible to address the individual needs of a particular animal. Readers are connected to the internet to process the data immediately for individual animals. The system recognizes individual properties of each animal and uses advanced technologies to optimize animal production. It enables the possibility to customize individual animal’s nutrient requirements.

Both the refinement of nutrient requirements and nutrigenomics can be considered as important elements toward the goal of precision nutrition in sheep and goats. The synergy between AI and nutrigenomics affords an important opportunity for the improvement of precision nutrition in small ruminants. Through AI algorithms, tailored nutritional strategies can be formulated based on the genetic profiles of animals. Application of AI in phenotyping compliments rapid development in genotyping such as pangenomics and nutrigenomics [132]. Applications of AI in precision nutrition include mass data collection and analysis and imaging, with the potential to reduce the production cost of large-scale operation in small ruminant production; these applications continue to evolve.

There are challenges in the application of AI to the prediction of nutrient requirements. It was pointed out that an important gap exists between data-driven AI models and the traditional concept- and knowledge-driven models that have been used to predict nutrient requirements in animals [133]. It remains to be realized if a hybrid intelligent mechanistic model as suggested can be adopted/developed to increase the precision in prediction of nutrient requirements in sheep and goats. For the application of AI to sheep farming, cost, lack of critical data mass, precision of predictions, ethical issues, and adaptability to various environments were identified as challenges [134]. The same challenges are expected in applying AI to goat production as well. Similar challenges were also suggested pertaining to the use of AI in Latin American ruminant production [135].

## 13. Conclusions

The update in nutrient requirements in sheep and goats merits considerations in light of advancements in research to the effects of species difference, diverse environment, genetic variations, and climate adaptation on nutrient requirements. Defining nutrient requirements will continue to be an important focus as a gateway to streamlining mass nutritional information and enabling the practical application to animals and will contribute to the simplification of complex nutrition research. Translating complex research in nutrigenomics into evidence-based practical guidelines is a challenge, and it remains to be seen for the eventual impact on recommendations of nutrient requirements. To conserve resources and maintain long-term sustainability, precision nutrition in the context of nutrient requirements will likely play a pivotal role. Meaningful research and discussion pertaining to implications of nutrigenomics and the application of AI to nutrient requirements in sheep and goats can be useful in defining nutritional needs of not only the population but also individuals.

## Figures and Tables

**Table 1:** Comparison of NRC [3] energy and protein requirements between sheep and goats.

Function	Species			ME Mcal/d	MP g/d
Maintenance	Sheep	Mature Ewes	50 kg BW	1.75	47
	Goat	Mature Dairy Doe	50 kg BW	2.25	53
		Mature Nondairy	50 kg BW	1.9	48
Growth	Sheep	Late Maturing	30 kg, 200 g/d	2.46	86
		Early Maturing	30 kg, 200 g/d	2.86	84
	Goat	Boer	30 kg, 200 g/d	2.49	120
		Dairy	30 kg, 200 g/d	2.74	97
Late Gestation	Sheep	Mature Ewes	50 kg, single	2.76	85
	Goat	Mature Dairy	50 kg, single	3.19	112
Early Lactation	Sheep	1.32 kg/d milk	50 kg, twin	3.85	170
		2.39 kg/d milk	90 kg, twin	5.54	237
	Goat	2.33 kg/d milk	50 kg, twin	4.41	205
		3.22 kg/d milk	90 kg, twin	6.61	297

## Data Availability

No new data were created or analyzed in this study. Data sharing not applicable.
